# Effects of Thyroid Hormone Analogue and a Leukotrienes Pathway-Blocker on Reperfusion Injury Attenuation after Heart Transplantation

**DOI:** 10.1155/2013/303717

**Published:** 2013-09-17

**Authors:** Fadhil G. Al-Amran, Najah R. Hadi, Haider S. H. Al-Qassam

**Affiliations:** Surgical Department, Medical College, Kufa University, Kufa Street, Najaf, Iraq

## Abstract

*Background*. Global myocardial ischemia reperfusion injury after heart transplantation is believed to impair graft function and aggravate both acute and chronic rejection episodes. *Objectives*. To assess the possible protective potential of MK-886 and 3,5-diiodothyropropionic acid DITPA against global myocardial ischemia reperfusion injury after heart transplantation. *Materials and Methods*. Adult albino rats were randomized into 6 groups as follows: group I sham group; group II, control group; groups III and IV, control vehicles (1,2); group V, MK-886 treated group. Donor rats received MK-886 30 min before transplantation, and the same dose was repeated for recipients upon reperfusion; in group VI, DITPA treated group, donors and recipients rats were pretreated with DITPA for 7 days before transplantation. *Results*. Both MK-886 and DITPA significantly counteract the increase in the levels of cardiac TNF-**α**, IL-1**β**, and ICAM-1 and plasma level of cTnI (*P* < 0.05). Morphologic analysis showed that both MK-886 and DITPA markedly improved (*P* < 0.05) the severity of cardiac injury in the heterotopically transplanted rats. *Conclusions*. The results of our study reveal that both MK-886 and DITPA may ameliorate global myocardial ischemia reperfusion injury after heart transplantation via interfering with inflammatory pathway.

## 1. Introduction

 Organ transplantation is a unique situation where grafts are successively subjected to global cold ischemia, warm ischemia, and blood reperfusion. These events are believed to impair graft function and aggravate both acute and chronic rejection episodes [1, 2]. The pathophysiology of ischemia/reperfusion (I/R) injury shows several characteristics of inflammatory responses including activation of complement, platelets, and endothelial cells, infiltration of monocytes and neutrophils, and the release of oxygen-derived-free radicals, chemokines, and cytokines [3]. MK-886 is a highly potent inhibitor of leukotriene formation *in vivo* and *in vitro* [4]. This compound inhibits leukotriene biosynthesis indirectly by a mechanism through the binding of a membrane-bound 5-lipoxygenase-activating protein (FLAP), thereby inhibiting the translocation and activation of 5-lipoxygenase [5, 6]. MK-886 was found to prevent both post ischemic leukotriene accumulation and the microcirculatory changes after ischemia-reperfusion [7]. MK-886 was also found to be effective in prevention of liver and intestine injury by reducing apoptosis and oxidative stress in a hepatic I/R model. Anti-inflammatory properties and inhibition of lipid peroxidation by MK-886 could be protective for these organs in (I/R) injury [8]. MK-886 inhibits early I/R-induced increase in intestinal P-selectin expression, where the selectins have been implicated in the recruitment of leukocytes into tissues exposed to (I/R) [9]. DITPA is a TH analog with low metabolic activity. It was identified as a compound of interest during the screening of thyromimetic compounds with low metabolic activity for their ability to induce a myosin heavy chain in fetal heart cells as an indication of their potential inotropic activities [10]. DITPA improved left ventricular performance in rabbit and rat postinfarction heart failure models when administered either alone or in combination with an angiotensin I-converting enzyme inhibitor [11]. DITPA can promote angiogenesis by interacting with membrane-bound integrin *α*V*β*3 and activating the MAPK cascade [12]. It also reduces infart size and attentuates the inflammatory response following myocardial ischemia [13].

## 2. Material and Methods

### 2.1. Animals and Study Design

 A total of 66 adult male albino rats weighing (180–220 g) were purchased from Animal Resource Center, National Center for Drug Control and Research. They were housed in the animal house of Kufa College of Medicine in a temperature-controlled (25° ± 1°C) room (humidity was kept at 60–65%) with alternating 12 h light/12 h dark cycles and were allowed free access to water and chow diet until the start of experiments. After the 1st week of acclimatization, the rats were randomized into 6 groups as follows.

- Sham group: rats underwent the same anesthetic and surgical procedures (for an identical period of time for global myocardial ischemia and reperfusion through transplantation) but with no heterotopic heart transplantation.
- Control group (induced untreated group): rats underwent 30 min of global myocardial ischemia followed by 60 min of reperfusion via heterotopic heart transplantation.
- Control vehicle (1) group: donors rats received vehicle of MK-886 (2% ethanol) i.p. 30 min before transplantation, and the same dose was repeated for recipients upon reperfusion.
- Control vehicle (2) group: donors and recipients rats pretreated with DITPA vehicle {0.1 N NaOH diluted with 0.9% saline (pH 9)} s.c. for 7 days before transplantation.
- MK-886 treated group: donors rats received MK-886 (0.6 mg/kg) i.p. injection 30 min before transplantation, and the same dose was repeated for recipients upon reperfusion.
- DITPA treated group: donors and recipients rats pretreated with DITPA (3.75 mg/kg) s.c. for 7 days before transplantation.

### 2.2. Drugs

#### 2.2.1. MK-886

The drug was purchased from (Santa Cruz, USA), and it was given in dose of 0.6 mg/kg dissolved in 2% ethanol via i.p. route. The drug was prepared immediately before use [8]. Ethanol was used to form a homogenized drug. Each dose was homogenized in 1 mL ethanol and injected via i.p [8].

#### 2.2.2. DITPA

 The drug was purchased from (Sigma, USA), and solution of DITPA was prepared immediately before use by dissolving the powder in 0.1 N NaOH and diluting with 0.9% saline (pH 9) [14].

### 2.3. Heterotopic Heart Transplantation by Using Cuff Method

 The rats were anesthetized with 100 mg/kg ketamine and 5 mg/kg xylazine [15] and placed in the supine position with their limbs immobilized and the skin of the operative region sterilized. Heterotopic heart transplantation was performed by a modified cuff technique previously described by Xiu et al. (2001) [16].

#### 2.3.1. Donor Operation

A midline abdominal incision was made in the donor, and 1 mL heparinized saline (100 U/mL) was injected into the inferior vena cava (IVC). Then, a bilateral thoracotomy was performed, and the anterior chest wall was opened. Blunt dissection of the aorta and pulmonary artery was performed. All the vessels, except the aorta and pulmonary artery, were ligated towards the posterior surface of the heart with a 4–0 silk suture. The donor heart was removed and stored in cold lactated ringer solution at 4°C [16].

#### 2.3.2. Recipient Operation

A longitudinal incision was made. The right submaxillary gland was removed to expose the right external jugular vein. The right common carotid artery was exposed by cutting the right sternocleidomastoid muscle. The proximal portion of the carotid artery was occluded with bulldog clamp and the distal portion of it was ligated with 6–0 silk. The carotid artery was then incised and the proximal end was irrigated with heparinized saline (100 U/mL). The carotid artery was then passed through the IV cuff; the proximal end of the carotid artery was everted over the cuff and wrapped with a circumferential ligature of 6–0 silk. The right external jugular vein was prepared in the same way. The donor heart was placed in the right neck of the recipient. The arterial cuff was inserted into donor aorta and fixed with a preset ligature (6–0 silk). An IV cuff was inserted into the donor pulmonary artery and also fixed with 6–0 silk ligature. The clamp on the external jugular vein was unclamped, followed by unclamping of the carotid artery. The heart rapidly turned from pale to red [16].

### 2.4. Preparation of Samples

#### 2.4.1. Blood Sampling for Measurement of Plasma cTnI

 At the end of the experiment, about 2 mL of blood as collected from the heart. The blood sample was placed in a tube containing disodium EDTA (22 mg/mL) as anticoagulant and mixed thoroughly then centrifuged at 3000 rpm for 15 min. Then it was used for determination of plasma cTnI according to the manufacturer's instructions and guidelines using enzyme-linked immunosorbent assay (ELISA) kits (Life Diagnostics, USA).

#### 2.4.2. Tissue Preparation for TNF-*α*, IL-1*β*, and ICAM-1 Measurement

 The basal side of the heart tissues was rinsed with ice cold saline to remove any red blood cells or clots and then homogenized with a high intensity ultrasonic liquid processor in 1 : 10 (w/v) phosphate buffered saline that contained 1% Triton X-100 and a protease inhibitor cocktail [17]. The homogenate was centrifuged at 2,500 g for 20 min at 4°C. The supernatant was collected for determination of TNF-*α*, IL-1*β*, and ICAM-1 according to the manufacturer's instructions and guidelines using enzyme-linked immunosorbent assay (ELISA) kits (RayBio, USA).

#### 2.4.3. Tissue Sampling for Histopathology

 At the end of the experiment, the transplanted hearts were excised and apical side of heart tissues fixed in 10% formalin and embedded in paraffin; the sections were stained with hematoxylin and eosin (H&E) after fixation. Evaluation scores were performed by an investigator who was blinded to the experimental treatment groups. The following morphological criteria were used to assess the histopathological damage: score 0, no damage; score 1 (mild), interstitial edema and focal necrosis; score 2 (moderate), diffuse myocardial cell swelling and necrosis; score 3 (severe), necrosis with the presence of contraction bands, neutrophil infiltration, and the compressed capillaries; and score 4 (highly severe), widespread necrosis with the presence of contraction bands, neutrophil infiltration, capillaries compressing, and hemorrhage [18].

### 2.5. Statistical Analysis

 Statistical analyses were performed by using SPSS 17.0 for windows.lnc. An expert statistical advice was consulted for tests used. Data were expressed as mean ± SEM. Analysis of variance (ANOVA) was used for the multiple comparisons among all groups followed by post hoc tests using LSD method. The statistical significance of difference in total score between 2 groups was assessed by Mann-Whitney *U* test. In all tests, *P* < 0.05 was considered to be statistically significant. 

## 3. Results

### 3.1. Effect on Proinflammatory Markers (TNF-*α*, IL-1*β*, and ICAM-1)

**Figure 1:**
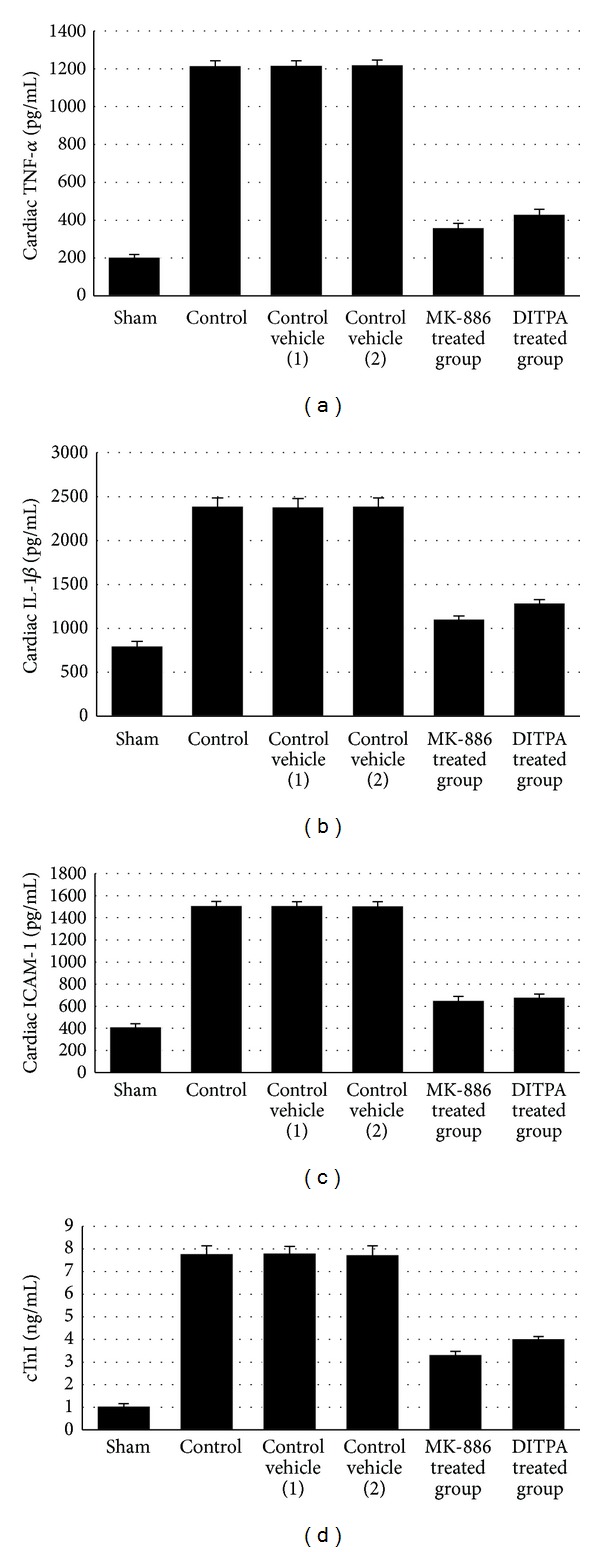
(a) The mean of cardiac TNF-*α* level (pg/mL) in the six experimental groups at the end of the experiment. (b) The mean of cardiac IL-1*β* level (pg/mL) in the six experimental groups at the end of the experiment (*N* = 6 in each group). (c) The mean of cardiac ICAM-1 level (pg/mL) in the six experimental groups at the end of the experiment. (d) The mean of plasma cTnI level (ng/mL) in the six experimental groups at the end of the experiment (number of animals = 6 in each group).

At the end of the experiment, the levels of cardiac TNF-*α*, IL-1*β*, and ICAM-1 were significantly (*P* < 0.05) increased in control group (II) as compared with the sham group (I). The levels of cardiac TNF-*α*, IL-1*β*, and ICAM-1 of MK-886 and DITPA treated groups were significantly (*P* < 0.05) lower than that of control vehicle (1) and (2) group, respectively. The values of cardiac TNF-*α*, IL-1*β*, and ICAM-1 are shown in Table 1 and Figures 1(a), 1(b), and 1(c).

### 3.2. Effect on Level of Plasma Cardiac Troponin I cTnI

At the end of the experiment, the level plasma of cTnI was significantly increased (*P* < 0.05) in control group (II) as compared with sham group (I). The plasma level of cTnI of MK-886 and DITPA treated group was significantly (*P* < 0.05) lower than that of control vehicle (1) and (2) group, respectively. The values of plasma levels of cTnI are shown in Table 2 and Figure 1(d).

### 3.3. Histopathological Findings

**Figure 2:**
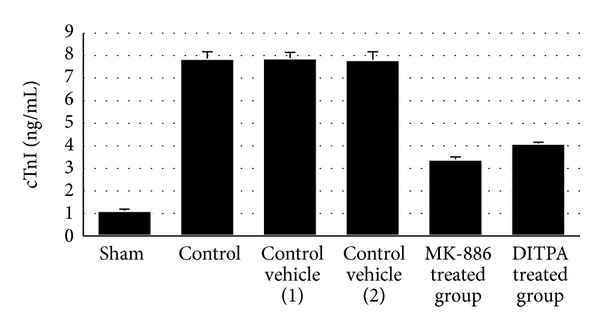
Error bar chart shows the difference in mean ± SEM values of total severity scores in the six experimental groups.

**Figure 3:**
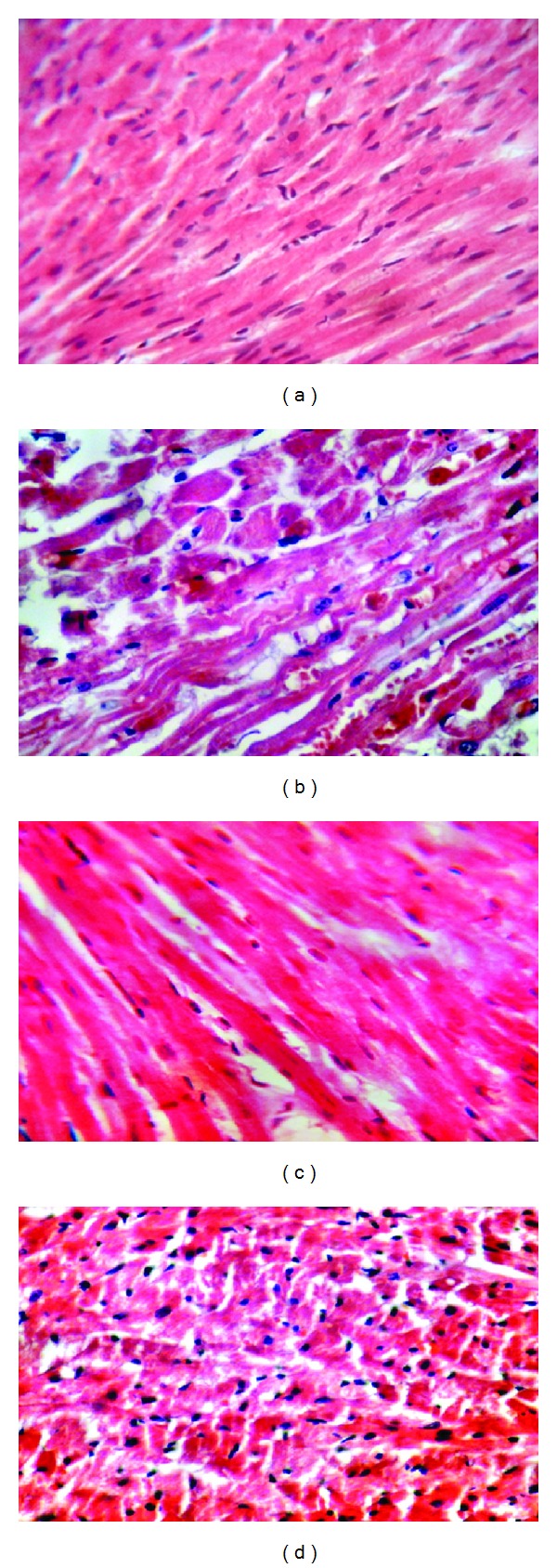
(a) Photomicrograph of cardiac section of normal rats shows the normal architecture. The section stained with haematoxylin and eosin (×40). (b) Photomicrograph of cardiac section showed extensive necrosis, contraction bands, and hemorrhage. The section stained with Haematoxylin and Eosin (×40). (c) Photomicrograph of cardiac section in MK-886 treated group. The section stained with Haematoxylin and Eosin (×40). (d) Photomicrograph of cardiac section in DITPA treated group. The section stained with Haematoxylin and Eosin (×40).

 A cross-section of sham rat's heart showed the normal cardiac structure, no interstitial edema and focal necrosis, no diffuse myocardial cell swelling and necrosis, no contraction bands, no neutrophil infiltration, no capillaries compressing, and no hemorrhage. All rats in this group showed normal heart 100%. There was statistically significant difference between control group (II) and sham group (I) (*P* < 0.05) and the total severity scores of the control group showed that 16.7% of the group had moderate cardiac injury, 66.7% had severe cardiac injury and 16.7% had highly severe cardiac injury. Treatment of rats with MK-886 improved cardiac injury significantly (*P* < 0.05) as compared with control vehicle (1) group, and the total severity scores mean of this group showed that 16.7% of the group had no damage, 66.7% had mild cardiac injury, and 16.7% had moderate cardiac injury. Treatment of rats with DITPA improved cardiac injury significantly (*P* < 0.05) as compared with control vehicle (2) group, and the total severity score mean of this group showed that 66.7% had mild cardiac injury and 33.3% had moderate cardiac injury as shown in Table 3 and Figures 2(a)–2(d) and Figure 3. 

## 4. Discussion

### 4.1. Effect of Global Myocardial Ischemia Reperfusion Injury after Heart Transplantation on Inflammatory Mediator (TNF-*α*)

In this study a significant increase in inflammatory mediator TNF-*α* level in cardiac tissues (*P* < 0.05) was found in control group as compared with sham group.

TNF-*α* is a proinflammatory cytokine that has been implicated in the pathogenesis of cardiovascular diseases, including I/R injury, heart failure, and cardiac allograft rejection [19]. Gurevitch et al. (1996) were the first to demonstrate a significant release of TNF-*α* in the rat coronary effluent at 1 min after reperfusion [20]. Meldrum et al. (1998) later demonstrated that TNF-*α* protein is elevated in the myocardium itself after crystalloid-perfused global ischemia-reperfusion [19, 21]. These studies indicate that the myocardium synthesizes and releases TNF-*α* in response to ischemia and reperfusion and consistent with this study. Myocardial ischemia induces degranulation of resident mast cells [22] and cleavage of membrane-bound TNF-*α* by the TNF-*α* cleavage enzyme (TACE) [23], both causing the immediate release of active TNF-*α* in the ischemic myocardium to act in an autocrine, endocrine, and paracrine fashion [24]. Reperfusion of ischemic myocardium imposes an oxidant burden in which the reduction product of molecular oxygen hydrogen peroxide and contributes to myocardial injury [25]. Hydrogen peroxide-induced activation of P38 MAP kinase may contribute to ischemia reperfusion-induced TNF-*α* production [26]. Oxidant stress also activates NF*κ*B, which may also play a role in the sequence of ischemia-reperfusion-induced TNF-*α* production.

### 4.2. Effect of Global Myocardial Ischemia Reperfusion Injury after Heart Transplantation on Inflammatory Mediator (IL-1*β*)

In our study the level of cardiac IL-1*β* was significantly increased (*P* < 0.05) in control group as compared with sham group.

 IL-1*β* plays a crucial role in mediating (I/R) injury after transplantation. Herskowitz et al. (1995) also demonstrated induced myocardial gene expression for IL-1*β* cytokine after permanent left anterior descending occlusion and temporary left anterior descending occlusion followed by reperfusion [27]. IL-1*β* induces the expression of adhesion molecules on endothelial cells, thus facilitating cellular infiltration. Furthermore it induces the production of prostaglandins through an increased expression of cyclooxygenases and increases the number of circulating neutrophils [28].

### 4.3. Effect of Global Myocardial Ischemia Reperfusion Injury after Heart Transplantation on Adhesion Molecule (ICAM-1)

In our study the level of cardiac ICAM-1 was significantly increased (*P* < 0.05) in control group as compared with sham group.

 Several studies revealed that ICAM-1 plays an important role in the early stages of cardiac damage following (I/R) injury. Kukielka et al. (1993) reported that ICAM-1 gene expression is regulated in reperfused canine myocardium. Low-level constitutive expression of ICAM-1 was present on vascular endothelium, but following 1 hr coronary occlusion and 1 hr reperfusion, ICAM-1 mRNA was detected in reperfused myocardium, and the mRNA levels were observed to increase further after 3 and 6 hr of reperfusion [29]. This study is in consistent with our finding. ICAM-1 on the endothelial cells mediates the firm adhesion of neutrophil to the endothelium [30]. *β*-integrin is the ligand for ICAM-1 and is expressed by neutrophils, monocytes, and macrophages [30]. The interaction between the endothelium and neutrophils activates neutrophils and their release of oxidant, protease, and thromboxane B2 and LTB4, which recruit other inflammatory cells and incur more damage to the heart [30].

### 4.4. Effect of Global Myocardial Ischemia Reperfusion Injury after Heart Transplantation on cTnI

 In present study the level of plasma cTnI was significantly increased (*P* < 0.05) in control group as compared with sham group.

Consistently Bertinchant et al. (1999) showed that cTnI was released in 1 min during 60 min of reperfusion after 20 min, 30 min, 40 min, or 60 min of global ischemia. cTnI peaked 1 min after reperfusion but did not return to baseline values and cTnI concentrations in the effluent increased throughout the reperfusion period by using isolated and Langendorff-perfused rat hearts model [31]. cTnI degradation during (I/R) involves both cleavage of cTnI and cross-linking between the troponin complex elements. On the basis of indirect evidence, the cleavage of cTnI has been attributed to calpain, a cysteine protease, while the cross-linking of troponin complex elements has been attributed to a cardiac tissue transglutaminase (TGase) isoform. Both of these enzymes are calcium dependent, which has led previous investigators to hypothesize that cTnI release reflects the intracellular accumulation of calcium [32, 33].

### 4.5. Effect of Global Myocardial Ischemia Reperfusion Injury after Heart Transplantation on Cardiac Tissue

 There was statistically significant difference between control group and sham group (*P* < 0.05), and the total severity scores of the control group showed that 16.7% of the group had moderate cardiac injury, 66.7% had severe cardiac injury, and 16.7% had highly severe cardiac injury. Consistently Zingarelli et al. (2002) showed that a marked disruption of the myocardial structure in myocardial (I/R) injury was characterized by appearance of extensive necrosis and contraction bands [34].

 In our study global myocardial (I/R) injury following heart transplantation was associated with increased levels of TNF-*α*, IL-1*β*, and ICAM-1 in cardiac tissues in addition to elevated plasma level of cTnI. After global myocardial ischemia, the first few minutes of reperfusion are of critical importance as lethal myocardial injury commences at this point. There is substantial evidence that neutrophils are recruited to the ischemic reperfused myocardium, where they release reactive oxygen species [35]. These species can have deleterious effect on the myocytes and endothelial cells thus causing myocardial injury. In addition, ischemic-reperfused myocytes release cytokines that can further activate neutrophils, thus enhancing reactive species generation and extending the infarcted area. The overwhelming production of proinflammatory mediators can lead to contractile dysfunction and cardiomyocyte death [36].

### 4.6. Effect of MK-886 on Inflammatory Mediators (TNF-*α*, IL-1*β*)

 In our study, MK-886 significantly reduced the elevation of inflammatory mediators (TNF-*α*, IL-1*β*) levels in cardiac graft tissues (*P* < 0.05) as compared with control group, suggesting that MK-886 has protective effect on global ischemia reperfusion injury following heart transplantation.

There is no data available about effect of MK-886 on TNF-*α* and IL-1*β* in global ischemia reperfusion injury by using heart transplantation model; however, in a study of other model Al-Amran et al. (2011) showed that MK-886 treatment significantly decreased serum TNF-*α* in hemorrhagic shock-induced acute lung injury [37].

### 4.7. Effect of MK-886 on Adhesion Molecule (ICAM-1)

 In our study, MK-886 significantly reduced the elevation of (ICAM-1) levels in cardiac graft tissues (*P* < 0.05) as compared with control group, suggesting that MK-886 has protective effect on global ischemia reperfusion injury following heart transplantation. To the best of our knowledge there is no study about effect of MK-886 on ICAM-1 in global ischemia reperfusion injury after heart transplantation, however Wang et al. (2004) found that MK-886 could inhibit the expression of ICAM-1 in human melanoma cells [38]. ICAM-1 is constitutively present on endothelial cells, but its expression is increased by proinflammatory cytokines [39]. Also leukotrienes can be synthesized by vascular endothelial cells, smooth muscle cells, neutrophils, macrophages, and platelets, and they can trigger the expression of adhesion molecules on the surface of endothelial cells and leukocytes [40]. Therefore inhibition of leukotrienes by MK-886 might result in lowering level of ICAM-1 in cardiac tissue.

### 4.8. Effect of MK-886 on Cardiac Tissue and cTnI

Treatment of rats with MK-886 improved cardiac injury significantly (*P* < 0.05) as compared with control group and the total severity scores mean of this group, showed mild cardiac injury.

 To the best of our knowledge there is no data available about effect of MK-886 on cardiac tissues. However Al-Amran et al. (2011) found that treatment of rats with MK-886 ameliorates the lung injury significantly (*P* < 0.05) as compared with induced untreated group, and the total score mean of this group shows mild lung injury in hemorrhagic shock-induced acute lung injury [37]. Daglar et al. (2009) found that MK-886 significantly reduces the histological changes in the liver and small intestine of rats that underwent hepatic (I/R) model [8]. 

 MK-886 significantly reduced the elevation of cTnI plasma level (*P* < 0.05) which was found in MK-886 treated group as compared with control group, but there is no data available about effect of MK-886 on cTnI. In present study we found that there is a significant strong positive correlation between level of cTnI and cardiac injury score (*r* = 0.862, *P* < 0.01), and treatment of rats with MK-886 ameliorates cardiac injury so it might result in lowering plasma level of cTnI. No study yet available about the effect of MK-886 on cTnI in this model to compare our results.

### 4.9. Effect of DITPA on Inflammatory Mediators (TNF-*α*, IL-1*β*)

 In the present study, DITPA significantly reduced the elevation of inflammatory mediators (TNF-*α*, IL-1*β*) levels in cardiac graft tissues (*P* < 0.05) as compared with control group, suggesting that DITPA has protective effect on global myocardial (I/R) injury following heart transplantation.

 To the best our knowledge there is no data about effect of DITPA treatment on TNF-*α* and IL-1*β* in global myocardial I/R injury following cardiac transplantation.

 One study explained that treatment with DITPA attenuates the acute inflammatory response following myocardial ischemia by reducing inflammatory mediators, IL-6 [31]. Another study revealed that inflammatory cytokines were inversely correlated with serum concentrations of thyroid hormones during pediatric cardiac surgery [40], so the treatment with DITPA might result in reducing levels of TNF-*α* and IL-1*β* in cardiac tissues.

### 4.10. Effect of DITPA on Adhesion Molecule (ICAM-1)

 In this study, DITPA significantly reduced the elevation of (ICAM-1) levels in cardiac graft tissues (*P* < 0.05) as compared with control group, suggesting that DITPA has a protective effect on global myocardial (I/R) injury following heart transplantation.

 To our knowledge there is no study about the effect of DITPA treatment on ICAM-1 level in global myocardial I/R injury following cardiac transplantation; however, Abohashem-Aly et al. (2011) showed that ICAM-1 was decreased by 50% in the ischemic myocardium of DITPA treated animals following myocardial ischemia [13]. ICAM-1 is an endothelial adhesion molecule important for transmigration of both neutrophils and macrophages into the heart [39, 40]. It can be induced on endothelial cells by pro-inflammatory cytokines such as TNF-*α* or IL-1*β* [33, 40]. In the present study, DITPA significantly reduced the elevation of inflammatory mediators (TNF-*α*, IL-1*β*) levels in cardiac graft tissues, so it might result in lowering levels of ICAM-1 in cardiac tissues.

### 4.11. Effect of DITPA on Cardiac Tissues and cTnI

 Treatment of rats with DITPA ameliorates cardiac injury significantly (*P* < 0.05) as compared with control group, and the total severity scores mean of this group showed mild cardiac injury.

There are studies in other models demonstrating a protective effect of DITPA in cardiac tissues. Abohashem-Aly et al. (2011) demonstrated that DITPA attenuates myocardial injury following permanent ischemia and that the cardioprotective effect of DITPA is associated with a reduction in postinfarction inflammation [13]. Ambrosio and Tritto (1999) have analyzed the effects of DITPA on myocyte morphology by treating normal and cardiomyopathic hamsters with DITPA for 2 months; they found that DITPA had beneficial effects on chamber remodeling in cardiomyopathic animals, which was likely due to beneficial changes in cell shape and improved vascular supply [36]. In the present study we found that DITPA treatment significantly (*P* < 0.05) reduced the increasing plasma levels of cTnI as compared with control group. Based on our knowledge there is no data available about effect of DITPA treatment on cTnI. In this study and others we observed that DITPA treatment attenuated myocardial injury, and also we know that cTnI is a specific marker for cardiac injury [40]; therefore, it might cause reduced levels of cTnI.

## Figures and Tables

**Table 1:** Cardiac TNF-*α*, IL-1*β*, and ICAM-1 levels (pg/mL) of the three experimental groups at the end of the experiment.

Group	TNF*α* (pg/mL)	IL-*β* (pg/mL)	ICAM-1 (pg/mL)
(1) Sham	205.79 ± 13.45	802.47 ± 48.37	414.35 ± 26.56
(2) Control	1217.01 ± 25.1*	2392.06 ± 90.62*	1512.26 ± 35.62*
(3) Control vehicle (1)	1219.03 ± 23.44	2385.39 ± 91.55	1512.46 ± 33.99
(4) Control vehicle (2)	1221.24 ± 25.97	2393.93 ± 88.73	1510.44 ± 35.39
(5) MK-886	361.63 ± 22.59^Ψ^	1109.8 ± 28.87^Ψ^	655.88 ± 33.04^Ψ^
(6) DITPA	431.24 ± 41^†^	1290.7 ± 35.88^†^	684.96 ± 25.03^†^

The data expressed as mean ± SEM (*N* = 6 in each group). *Versus sham group, ^Ψ^versus control vehicle (1) group, ^†^versus control vehicle (2).

**Table 2:** 

Groups	cTnI (ng/mL)
(1) Sham	1.06 ± 0.1
(2) Control	7.81 ± 0.33*
(3) Control vehicle (1)	7.83 ± 0.28
(4) Control vehicle (2)	7.76 ± 0.37
(5) MK-886	3.34 ± 0.13^Ψ^
(6) DITPA	4.04 ± 0.09^†^

The data expressed as mean ± SEM (*N* = 6 in each group). *Versus sham group, ^Ψ^versus control vehicle (1) group, ^†^versus control vehicle (2).

**Table 3:** The differences in histopathological scoring of abnormal heart changes among the six experimental groups.

Histopathological scoring	Study groups											
	Sham		Control		Control vehicle (1)		Control vehicle (2)		MK-886		DITPA	
	*N*	%	*N*	%	*N*	%	*N*	%	*N*	%	*N*	%
Score 0 (no damage)	6	100	0	0	0	0	0	0	1	16.7	0	0
Score 1 (mild)	0	0	0	0	0	0	0	0	4	66.7	4	66.7
Score 2 (moderate)	0	0	1	16.7	0	0	2	33.3	1	16.7	2	33.3
Score 3 (severe)	0	0	4	66.7	5	83.33	3	50	0	0	0	0
Score 4 (highly severe)	0	0	1	16.7	1	16.7	1	16.7	0	0	0	0

Total	6	100	6	100	6	100	6	100	6	100	6	100
